# Hepatoprotective and Antioxidant Effects of Saponarin, Isolated from *Gypsophila trichotoma* Wend. on Paracetamol-Induced Liver Damage in Rats

**DOI:** 10.1155/2013/757126

**Published:** 2013-06-26

**Authors:** Rumyana Simeonova, Vessela Vitcheva, Magdalena Kondeva-Burdina, Ilina Krasteva, Vassil Manov, Mitka Mitcheva

**Affiliations:** ^1^Laboratory of Drug Metabolism and Drug Toxicity, Department of Pharmacology, Pharmacotherapy and Toxicology, Faculty of Pharmacy, Medical University, 2 Dunav Street, 1000 Sofia, Bulgaria; ^2^CFSAN, US FDA, 5100 Paint Branch, Parkway College Park, MD 20740, USA; ^3^Department of Pharmacognosy, Faculty of Pharmacy, Medical University, 2 Dunav Street, 1000 Sofia, Bulgaria; ^4^Department of Obstetrics, Gynecology, Biotechnology of Reproduction, Pathological Anatomy and Biochemistry, Faculty of Veterinary Medicine, University of Forestry, 10 Kliment Ohridski Boulevard, 1756 Sofia, Bulgaria

## Abstract

The hepatoprotective potential of saponarin, isolated from *Gypsophila trichotoma*, was evaluated *in vitro/in vivo* using a hepatotoxicity model of paracetamol-induced liver injury. In freshly isolated rat hepatocytes, paracetamol (100 **μ**mol) led to a significant decrease in cell viability, increased LDH leakage, decreased levels of cellular GSH, and elevated MDA quantity. Saponarin (60–0.006 **μ**g/mL) preincubation, however, significantly ameliorated paracetamol-induced hepatotoxicity in a concentration-dependent manner. The beneficial effect of saponarin was also observed *in vivo*. Rats were challenged with paracetamol alone (600 mg/kg, i.p.) and after 7-day pretreatment with saponarin (80 mg/kg, oral gavage). Paracetamol toxicity was evidenced by increase in MDA quantity and decrease in cell GSH levels and antioxidant defence system. No changes in phase I enzyme activities of AH and EMND and cytochrome P 450 quantity were detected. Saponarin pretreatment resulted in significant increase in cell antioxidant defence system and GSH levels and decrease in lipid peroxidation. The biochemical changes are in good correlation with the histopathological data. Protective activity of saponarin was similar to the activity of positive control silymarin. On the basis of these results, it can be concluded that saponarin exerts antioxidant and hepatoprotective activity against paracetamol liver injury *in vitro/in vivo*.

## 1. Introduction

Liver disease is a serious medical problem. Some of the liver injuries are caused by the use and abuse of drugs. Conventional and/or synthetic drugs such as steroids, vaccines, antivirals, and other medications can cause serious side effects, even toxic effects on the liver, especially when used for prolonged periods of time [1]. There is a global trend towards the use of traditional herbal preparations for the treatment of liver diseases. The list of hepatoprotective biologically active compounds (BAC) in the scientific literature is quite long, but only some of them have enough strong effects to combat different types of liver damage. Some of them such as silymarin and curcumin have attracted the attention of the scientific community [2]. Many of these BAC are flavonoids, which are known to have various biological and pharmacological effects, for example, antibacterial, antiviral, antioxidant, and antimutagenic effects [3]. 

The genus *Gypsophila L.* (Caryophyllaceae) comprises about 150 species and six of them are found in Bulgaria. Gypsophila species are well known by their medicinal, decorative, and industrial applications. Some of Gypsophila species are used for medical treatment purposes, as drug in response to certain diseases such as an expectorant and diuretic, for treatment of hepatitis, gastritis, and bronchitis. Different Gypsophila species can be used as drugs for bone deformations, rheumatism, pimples, skin diseases, bile disorders, and liver problems [4].

*Gypsophila trichotoma* Wend. (Caryophyllaceae) is a perennial herbaceous plant located in southeast Europe, southwest Asia, Kazakhstan, west Mongolia, Russia, and Turkmenistan. In Bulgaria, the plant is found along the Black Sea coast [5]. The species is protected by the National Biodiversity Act. Our previous phytochemical studies of *G. trichotoma* showed the presence of triterpene saponins, flavonoids, sterols, and volatile compounds [6–8]. Saponarin, a naturally occurring apigenin-6-C-glucosyl-7-O-glucoside, is the main flavone glycoside isolated in a high amount (2 g) from *G. trichotoma*, for which preliminary *in vitro* [9] antioxidant activity was evaluated. Saponarin has been reported to possess hypoglycemic, antimicrobial, and hepatoprotective properties [10–12].

Hepatoprotective activity of different flavonoids has been demonstrated by various researchers against experimental models of hepatotoxicity. Silymarin, a flavonolignan from “milk thistle” (*Silybum marianum*) plant, is used almost exclusively for hepatoprotection due to its antioxidant, antilipid peroxidant, antifibrotic, and anti-inflammatory properties [13]. Silymarin offers good protection in various toxic models of experimental liver diseases, induced by acetaminophen, carbon tetrachloride, ethanol, D-galactosamine, and *Amanita phalloides *toxin [13]. The well-known pharmacological properties together with the well-studied mechanism of action make silymarin a preferable control compound especially in experimental studies with biologically active substances which mechanisms are still to be clarified. 

Paracetamol, a widely used over-the-counter (OTC) analgesic and antipyretic, is one of the best known experimental models of hepatotoxicity [14]. It is safe at therapeutic doses but causes a fatal hepatic necrosis and hepatic failure in overdose [15]. It was found that CYP2E1, CYP1A2, CYP3A4, depletion of intracellular GSH, and oxidative stress are the major mechanisms involved in the pathogenesis of paracetamol-induced liver injury [16]. 

Many research efforts are directed to the discovery and development of agents, which might protect cells from oxidative reactions with potential antioxidant and hepatoprotective effects [17].

Based on these data, the present study aims to trace the antioxidant and hepatoprotective effects of saponarin isolated from *Gypsophila trichotoma* on paracetamol-induced hepatotoxicity and to compare its effects with those of the classic antioxidant and hepatoprotector silymarin. 

## 2. Materials and Methods

### 2.1. Plant Material, Extraction, and Isolation of Saponarin

Plant material (overground parts) was collected in August 2008 at the Black Sea coast, Bulgaria. A voucher specimen (SO 103887) was deposited at the Herbarium of Faculty of Biology, Sofia University. ^1^H NMR (400 MHz) and ^13^C NMR (100.6 MHz) spectra were recorded on Bruker DPX-400 and Bruker AMX-400. HR-EIMS was carried out Varian MAT CH7A. Thin layer chromatography (TLC) study was carried out on silica gel plates (Kieselgel G, F254, 60, Merck) with solvent systems *n*-BuOH-AcOH-H_2_O (4 : 1 : 1), CHCl_3_/MeOH (9 : 1), EtOH/NH_3_/H_2_O (80 : 4 : 16). The spots were visualised by NTS/PEG reagent (for flavonoids) and anisidine/hydrogen phthalate reagent, followed by heating at 110°C (for sugars). Column chromatography was carried out with Diaion HP20. Acid hydrolysis was performed with 7% methanolic HCl for 3 h. 

**Figure 1:**
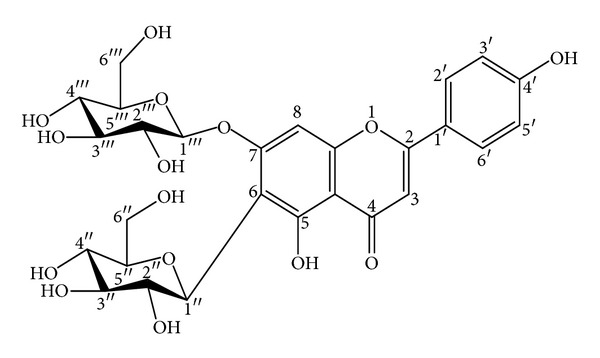
Structure of saponarin.

Air-dried powdered plant material (740 g) was exhaustively extracted with 80% methanol. After partial evaporation the aqueous solution was extracted with CH_2_Cl_2_, EtOAc, and *n*-BuOH, successively. The residue from the *n*-BuOH layer was separated on a Diaion HP 20 column, using water-methanol (H_2_O-MeOH) (0 → 100%) to yield saponarin (apigenin 6-C-glucosyl-7-O-glucoside) (2 g) (Figure 1). HPLC analysis shows 98% purity of saponarin. Structural assessment of the compound was effected by acid hydrolysis and analysis of MS (ESI-MS: 595.1690 [M+H]^+^) and ^1^H and ^13^C NMR spectroscopic data [9]. The *in vitro* antioxidant activity of saponarin was evaluated by a DPPH test (88.8% inhibition of DPPH radical in the concentration range 0.5 mg/mL) [9]. For the following *in vitro*/*in vivo* experiments, saponarin was dissolved, *ex tempore*, in redistilled water by sonification. 

### 2.2. Animals

Male Wistar rats (body weight 200–250 g) were used. The rats were housed in plexiglass cages (3 per cage) in a 12/12 light/dark cycle, under standard laboratory conditions (ambient temperature 20°C ± 2°C and humidity 72% ± 4%) with free access to water and standard pelleted rat food 53–3, produced according ISO 9001:2008. Animals were purchased from the National Breeding Center, Sofia, Bulgaria. A minimum of 7-day acclimatization was allowed before the commencement of the study and their health was monitored regularly by a veterinary physician. Vivarium (certificate of registration of farm No. 0072/01.08.2007) was inspected by the Bulgarian Drug Agency in order to check the husbandry conditions (No. A-11-1081/03.11.2011). All performed procedures were approved by the Institutional Animal Care Committee and the principles stated in the European Convention for the Protection of Vertebrate Animals used for Experimental and other Scientific Purposes (ETS 123) [18] were strictly followed throughout the experiment.

### 2.3. Chemicals

All the reagents used were of analytical grade. Paracetamol, silymarin, as well as other chemicals, Collagenase, 1-chloro-2,4-dinitrobenzene, beta-Nicotinamide adenine dinucleotide 2′-phosphate reduced tetrasodium salt (NADPH), ethylenediaminetetraacetic acid (EDTA), bovine serum albumin (fraction V), 2,2′-dinitro-5,5′dithiodibenzoic acid (DTNB) obtained from MERCK (Germany) reduced glutathione GSH), oxidized glutathione (GSSG), glutathione reductase (GR), and cumene hydroperoxide were purchased from Sigma Chemical Co. (Taufkirchen, Germany). 2,2′-Dinitro-5,5′dithiodibenzoic acid (DTNB) was obtained from Merck (Darmstadt).

### 2.4. Isolation and Incubation of Hepatocytes

Rats were anesthetized with sodium pentobarbital (0.2 mL/100 g). *In situ *liver perfusion and cell isolation were performed as described by Fau et al. [19], with modifications [20]. Cells were counted under the microscope and cell viability was assessed by Trypan blue exclusion (0.05%) [19]. Initial viability averaged 89%. 

#### 2.4.1. Methods for Assessing the Functional and Metabolic Status of Hepatocytes

Liver damage was induced by one hour incubation of the isolated hepatocytes with paracetamol (100 *μ*mol). In order to investigate the hepatoprotective activity of saponarin, the hepatocytes were preincubated for 30 min with the compound, administered in four concentrations (60–0.006 *μ*g/mL), and then incubated with paracetamol (100 *μ*mol) for one hour. The effect of saponarin was compared to those of silymarin (50–0.005 *μ*g/mL). The following parameters were measured to assess the functional status of hepatocytes: cell viability, lactate dehydrogenase (LDH) activity, reduced glutathione (GSH) levels, and malondialdehyde (MDA) quantity. Cell viability was assessed by Trypan blue exclusion method [19]. The dye was used at a final concentration of 0.05% and cells were counted under light microscope (×100). At the end of incubation, the cells were recovered via centrifugation at 400 ×g at 4°C. The supernatant was used for LDH and MDA assessment as described by Bergmeyer et al. [21] and Fau at al. [19], respectively. GSH measurement following the method used by Fau at al. [19] was assessed in the sediment. 

### 2.5. Design of the *In Vivo* Experiment

The animals were divided into six groups (*n* = 6). Saponarin and silymarin were administered daily by gavage at a dose volume of 5 mL/kg bw.

 Group 1: control animals, treated with the vehicle administered by gavage at 5 mL/kg bw/day.
 Group  2: treated with saponarin alone (80 mg kg^−1^/oral-gavage/7 days/week) [10].
 Group 3: treated with silymarin (100 mg kg^−1^/oral-gavage/7 days/week) [22].
 Group 4: up to day 7 the animals were treated the same way as groups 2 and 3 but with the vehicle only and on day 7 were they challenged with paracetamol (600 mg/kg i.p. once) [23].
 Group 5: treated with saponarin (80 mg kg^−1^/oral-gavage/7 days/week), 90 minutes after the last treatment challenged with paracetamol (600 mg/kg i.p.).
 Group 6: treated with silymarin (100 mg kg^−1^/oral-gavage/7 days/week), 90 minutes after the last treatment challenged with paracetamol (600 mg/kg i.p.).

The animals in all groups were sacrificed on the eighth day of the beginning of the experiment. Livers were taken for assessment of biochemical parameters. For all following experiments the excised livers were washed out with cold saline solution (0.9% NaCl), blotted dray, weighed, and homogenized with appropriate buffers. 

### 2.6. Preparation of Liver Homogenate for Lipid Peroxidation (LPO) Assessment

Lipid peroxidation was determined by measuring the rate of production of thiobarbituric acid reactive substances (TBARS) (expressed as malondialdehyde (MDA) equivalents) described by Polizio and Peña [24] with slight modifications. Briefly one volume of homogenate was mixed with 1 mL 25% trichloroacetic acid (TCA) and 1 mL 0.67% thiobarbituric acid (TBA). Samples were then mixed thoroughly, heated for 20 min in a boiling water bath, cooled and centrifuged at 4000 rpm for 20 min. The absorbance of supernatant was measured at 535 nm against a blank that contained all the reagents except the tissue homogenate. MDA concentration was calculated using a molar extinction coefficient of 1.56 × 10^5^ M^−1 ^cm^−1^ and expressed in nmol/g wet tissue.

### 2.7. Preparation of Liver Homogenate for GSH Assessment

GSH was assessed by measuring nonprotein sulfhydryls after precipitation of proteins with TCA, using the method described by Bump et al. [25]. Briefly, tissues were homogenized in 5% trichloroacetic acid (TCA) and centrifugated for 20 min at 4 000 ×g. The reaction mixture contained 0.05 mL supernatant, 3 mL 0.05 M phosphate buffer (pH = 8), and 0.02 mL DTNB reagent. The absorbance was determined at 412 nm and the results expressed as nmol/g wet tissue.

### 2.8. Preparation of Liver Microsomes for Biochemical Assay

The excised, perfused, and minced livers were homogenized with 3 mL of 1.17% KCl than centrifugated at 10 000 ×g for 30 min. The supernatant fractions were further centrifugated at 105 000 ×g for 60 min. The resulting microsomal pellets were stored at −20°C until assayed. At the day of assay the microsomal pellets were resuspended and diluted with phosphate buffer EDTA (pH = 7.4) [26]. Liver protein concentration was measured, using the method of Lowry [27] and was adjusted to 4 mg/mL.

#### 2.8.1. Evaluation of Phase I of Biotransformation

*(1) Assessment of Cytochrome P450 Quantity.* Cytochrome P450 was quantified spectrophotometrically as a complex with CO at 450 nm and expressed as nmol/mg^−1^ protein [28].

*(2) Assay of Aniline 4-Hydroxylase Activity (AH). *The method used 4-hydroxylation of aniline to 4-aminophenol that is chemically converted to a phenol-indophenol complex with an absorption maximum at 630 nm. Enzyme activity was expressed as nmol/min/mg [29].

*(3) Assay of Ethylmorphine-N-Demethylase (EMND Activity).* The enzyme activity was evaluated by the formation of formaldehyde, trapped in the solution as semicarbazone, and measured by the colorimetric procedure of Nash, at 415 nm. Enzyme activity was expressed as nmol/min/mg [29]. 

#### 2.8.2. Preparation of Liver Homogenates for Antioxidant Enzyme Activity Measurement

The livers were rinsed in ice-cold physiological saline and minced with scissors. A total of 10% homogenates were prepared in 0.05 M phosphate buffer (pH = 7.4) and centrifuged at 7,000 ×g and the supernatant was used for antioxidant enzymes assay. The protein content of liver homogenate was measured by the method of Lowry [27]. 

Catalase (CAT) activity was determined by measuring the decrease in absorbance at 240 nm. The enzyme activity was expressed as *μ*M/mg [30]. Superoxide dismutase activity (SOD) was measured according to the method of Misura and Fridovich [31]. SOD activity was expressed as nmol of epinephrine that are prevented from autoxidation after addition of the sample. Gluthatione peroxidase activity (GPx) was measured by NADPH oxidation, using a coupled reaction system consisting of glutathione, glutathione reductase, and cumene hydroperoxide [32]. The rate of disappearance of NADPH with time was determined by monitoring absorbance at 340 nm. Results are expressed in nmol/mg. 

Glutathione reductase activity (GR) was measured according to the method of Pinto et al. [33] by following NADPH oxidation spectrophotometrically at 340 nm and using an extinction coefficient of 6.22 mM^−1 ^cm^−1^. Glutathione-S-transferase activity (GST) was measured using 1-chloro-2,4-dinitrobenzene (CDNB) as substrate [34]. The enzyme activity is expressed as nmol of CDNB-GSH conjugate formed/min/mg protein. 

### 2.9. Histopathological Studies

For light microscopic evaluation, liver tissues were fixed in 10% buffered formalin and then thin sections (4 *μ*m) were subsequently stained with hematoxylin/eosin for general histological features determination [35]. Sections were studied under light microscope Leica DM 500.

## 3. Statistical Analysis

Statistical programme “MEDCALC” was used for analysis of the data. For the *in vitro* experiments the results are expressed as mean ± SEM of four animals per group and for each of the examined parameters, three parallel samples were used. For the *in vivo* experiments the data are expressed as mean ± SEM of six rats in each group. The significance of the data was assessed using the nonparametric Mann-Whitney test. For both statistical methods, values of *P* ≤ 0.05 were considered statistically significant.

## 4. Results

### 4.1. *In Vitro* Studies on Isolated Hepatocytes

Table 1 represents the effects of the first series of experiments in which hepatocytes were incubated with saponarin and silymarin in four decreasing concentrations. 

Both compounds, administered alone at the highest concentration, showed some hepatotoxic effects as follows: saponarin decreased cell viability by 36% (*P* < 0.05), decreased GSH levels by 45% (*P* < 0.05), increased LDH leakage into the medium by 82% (*P* < 0.05), and the amount of MDA by 76% (*P* < 0.05). Silymarin decreased cell viability by 47% (*P* < 0.05), depleted GSH levels by 55% (*P* < 0.05), and increased LDH leakage by 88% (*P* < 0.05) and the amount of MDA by 94% (*P* < 0.05). The results were compared to non-treated control hepatocytes.

**Figure 2:**
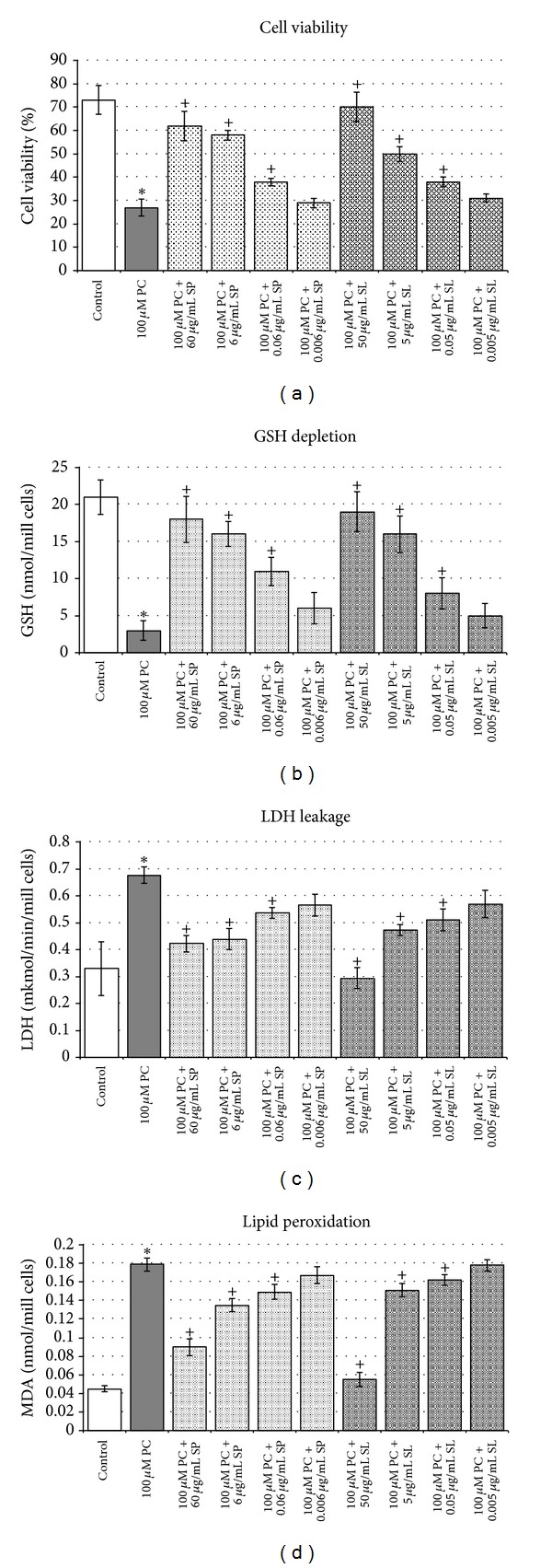
Effects of silymarin and saponarin preincubation on paracetamol-induced model of toxicity in isolated rat hepatocytes. Data are expressed as mean ± SD of four different experiments. *Significant difference from control values (Mann-Whitney test, *P* < 0.05). ^+^Significant difference from paracetamol-treated group (Mann-Whitney test, *P* < 0.05).

Figure 2 depicts the effects of paracetamol alone and the influence of saponarin and silymarin preincubation on paracetamol-induced hepatic damage. Compared to non-treated control hepatocytes, paracetamol induced hepatotoxicity, judged by: reduced cell viability by 63% (*P* < 0.05) (Figure 2(a)), depleted GSH levels by 86% (*P* < 0.05) (Figure 2(b)), increased LDH activity by 106% (*P* < 0.005) (Figure 2(c)) and nearly four times elevated MDA quantity (Figure 2(d)). 

Both saponarin and silymarin prevented the liver injury as judged by the preserved cell viability and the restored LDH activity, MDA quantity and GSH levels in a concentration-dependent manner as the effects were the most pronounced at the highest concentration. However, at this model of toxicity silymarin had better cytoprotective effects compared to saponarin. 

### 4.2. *In Vivo* Experiments

During the study no signs of toxicity were observed. All animals survived till the end of the treatment period.

#### 4.2.1. Lipid Peroxidation, Cell Glutathione, and Antioxidant Enzymes

Effect of saponarin and silymarin pretreatment on lipid peroxidation and antioxidant profile in rats with induced paracetamol toxicity is shown in Table 2.

*(1) Malondialdehyde Level in the Rat Liver Homogenate.* In paracetamol-treated rats the MDA level was increased by 30% (*P* < 0.05). Compared to PC-treated group pretreatment with saponarin led to a significant reduction of MDA by 18% (*P* < 0.05). In this toxic model MDA content was restored to the control level as by saponarin, as well as by silymarin.

*(2) GSH Level in the Rat liver Homogenate. *Compared to control animals, paracetamol led to a severe reduction in GSH levels by 83% (*P* < 0.05). Pretreatment with saponarin and silymarin resulted in significantly higher levels of GSH, compared to the group treated only with the respective hepatotoxic agent.

*(3) Activities of Antioxidant Enzymes SOD, CAT, GPx, GR, and GST. *Compared to control animals, acute administration of paracetamol led to significant decrease (*P* < 0.05) in SOD activity by 35%, in CAT activity by 27%, in GPx activity by 27%, in GR activity by 38%, and in GST activity by 31%. Pretreatment with saponarin for seven days and a subsequent single i.p. administration of paracetamol on the seventh day produced significant (*P* < 0.05) increase in SOD activity by 24%, in GPx activity by 21% in GR activity by 39%, and in GST activity by 21%, compared to the group treated with paracetamol alone. 

#### 4.2.2. Phase I of Biotransformation

In Table 3 the effects of the tested compounds administered alone and in combination on EMND and AH activities and cytochrome P 450 quantity are shown. Administered alone, paracetamol did not show any effect on the investigated parameters. The known inhibitory effect of silymarin is observed and in our study. Saponarin alone also showed some inhibitory effect, witnessed by decrease in total P450 quantity by 25% (*P* < 0.05) and decreased in EMND and AH activity by 24% (*P* < 0.05) and by 37% (*P* < 0.05), respectively. This inhibitory effect of the compounds was registered also in the combination groups.

#### 4.2.3. Histopathology

**Figure 3:**
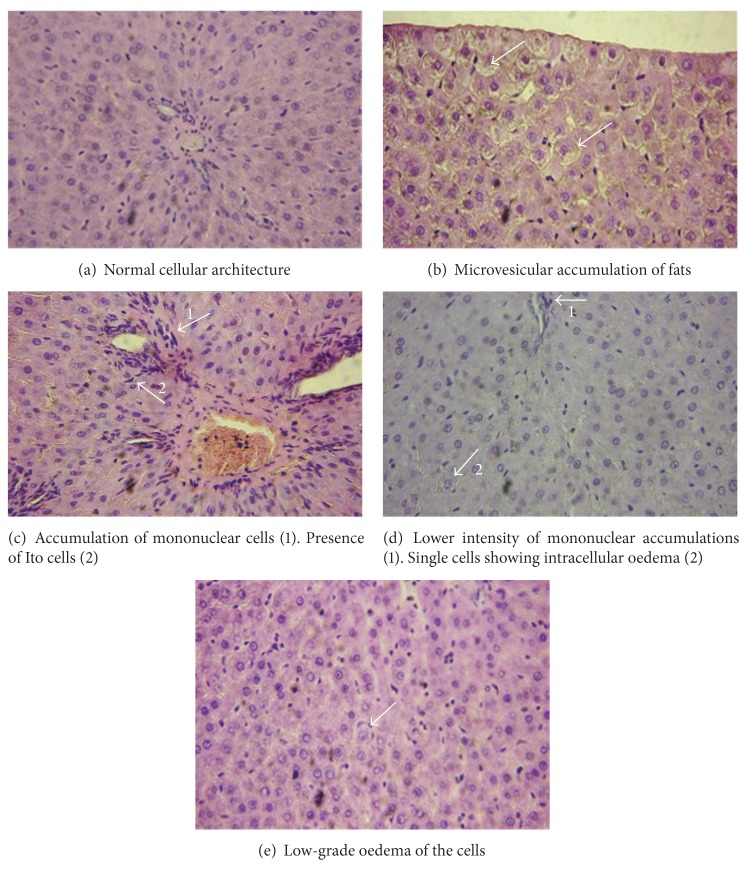
Histopathological profile. (a) Liver tissue from control rats revealed normal cellular architecture (magnification 400x). (b) Liver tissue from paracetamol-treated group revealed a presence of centriacinar and microvesicular accumulation of fats (magnification 400x). (c) Liver tissue from paracetamol-treated group revealed accumulation of mononuclear cells and reaction of the connective tissue around the bile ducts. Hyperplasia of cholangiocytes and presence of Ito (magnification 400x). (d) Liver tissue from rats pretreated with saponarin and then challenged with paracetamol showing lower intensity of mononuclear accumulations and the presence of single cells showing intracellular oedema (magnification 400x). (e) Liver tissue from rats pretreated with silymarin and then challenged with paracetamol low-grade oedema of the cells (magnification 400x).

The microscopic examination of the livers obtained from paracetamol-treated rats revealed treatment-related changes associated with hepatotoxicity. Histopathologically the changes in the liver were characterized by swelling of cells located near the liver capsule and around the terminal hepatic venule, and lytic changes in the nuclei and cytoplasm of the hepatocytes and pyknotic changes in the nuclei of some cells occurred (Figure 3(b)). A concentration of mononuclear cells and single leukocytes among hepatic parenchyma and their perivascular accumulation to terminal hepatic venules and the vessels of the portal triad were observed. In the portal triad were observed hyperplasia of cholangiocytes and availability of Ito cells (Figure 3(c)). Liver tissue from rats pretreated with saponarin and then challenged with paracetamol showed lower intensity of mononuclear accumulations and the presence of single cells showing intracellular oedema (Figure 3(d)). Liver tissue from rats pretreated with silymarin and then challenged with paracetamol showed low-grade oedema of the cells (Figure 3(e)).

## 5. Discussion 

Metabolism of chemicals takes place largely in the liver, which accounts for the organ's susceptibility to metabolism-dependent drug-induced injury. Drug-induced liver injuries are widespread and account for approximately one-half of the cases of acute liver failure and mimics all forms of acute and chronic liver disease [36]. Most of the drugs or their metabolites associated with hepatic damage induce their hepatotoxicity by interfering with the cell antioxidant systems causing free radical formation and oxidative stress initiation. Natural plant-derived products containing mostly flavonoids are being investigated as a source of antioxidants as these may have great relevance in the prevention of diseases associated with oxidative stress [37]. Hepatoprotective and antioxidant effects of flavonoids are well known both in experimental and in clinical practice. Silymarin, a mixture of polyphenolic flavonoids, derived from the fruits and seeds of Silybum marianum (milk thistle) is one of the most commonly used in medical practice hepatoprotective and antioxidant drug [38]. Along with it, however, identifying new sources of compounds with potent antioxidant and hepatoprotective activity is considered to be of great importance for the treatment of drug-induced liver injuries. 

The aim of the following study was to elucidate the possible mechanisms of protection of saponarin, a naturally occurring apigenin-6-C-glucosyl-7-O-glucoside, isolated from *Gypsophila trichotoma *Wed., against paracetamol-induced hepatotoxicity *in vitro/in vivo *using Wistar rats.

In experimental toxicology paracetamol-induced liver injury is used as a model of hepatotoxicity both *in vitro *and *in vivo*. The basic mechanism of paracetamol toxicity in the liver is well known and is related to the covalent binding of its reactive metabolite N-acetyl-p-benzoquinone imine (NAPQI) to sulfhydryl groups of GSH and various thiol-containing proteins and their subsequent oxidation [16]. Thus GSH depletion is considered one of the main biochemical markers for paracetamol-caused hepatotoxicity. Furthermore, the depletion of GSH causes the endogenous reactive oxygen species (ROS) to bind to cellular macromolecules leading to initiation of processes of lipid peroxidation, membrane breakdown, and cell death [39]. In our experiments, paracetamol caused statistically significant increase in MDA quantity and depletion in GSH levels, both *in vitro* and *in vivo*. In isolated hepatocytes these changes are accompanied by increased membrane permeability, evidenced by an enhanced leakage of LDH into the medium and a decrease in hepatocytes viability (see Figure 2). However, preincubation of the isolated hepatocytes with saponarin showed a concentration-dependent hepatoprotective activity, manifested by increase in cell viability, GSH levels, decrease in LDH activity, and MDA quantity, in comparison to paracetamol-only incubated hepatocytes (Figure 2). This beneficial effect of both saponarin and silymarin could be due to their antioxidant and membrane stabilizing properties, as well as to the possible influence on paracetamol metabolism, a suggestion that is supported by our *in vivo* results on phase I biotransformation enzymes taking part in paracetamol bioactivation. 

Regarding the mechanism of paracetamol toxicity, as well as the suggested antioxidant effect of saponarin, the antioxidant enzyme GST, GPx, GR, SOD, and CAT activities were registered in our *in vivo* series of experiments. As it was expected, in the paracetamol only group, the severe GSH depletion by 83% caused disturbances in the antioxidant defense, judged by significant reduction, by 31-32%, in GSH-depleting enzymes GPx and GST. GSH-replenishing enzyme—GR—was reduced by 38% (see Table 2). Paracetamol-induced biochemical disturbances are supported by the histological changes observed in livers obtained from paracetamol-challenged animals: swelling, mononuclear cells and single leukocytes cellular infiltration and hyperplasia of cholangiocytes and availability of Ito cells. In the group pretreated with saponarin and then challenged with paracetamol our data demonstrated significant hepatoprotective and antioxidant effect of saponarin as prevented the effects of paracetamol, observed on liver lipid peroxidation, GSH, and antioxidant defense system (see Table 2). Histopathological examination revealed a significant improvement of hepatocellular architecture (see Figure 3).

The antioxidant mechanism of saponarin includes scavenging of free radicals (DPPH test) [9]. It has also been suggested that saponarin acts as an antioxidant also by inhibiting oxidative enzymes such as xanthine oxidase (an important biological source of superoxide radicals) and have superoxide scavenging activity [3]. Therefore, the apparent hepatoprotective effect might be due to the ability of saponarin to neutralize the increase of free radicals caused by paracetamol. Furthermore, we suggested that saponarin might exert its hepatoprotective effects trough modulation of hepatic biotransformation. Several studies have found polyphenols to interact with cellular defense systems such as phases I (mainly the CYP450 complex enzymes) and II (e.g., glutathione transferases and glucuronyl transferases) detoxifying enzymes [40]. It is well known that plant sources containing flavonoids (e.g., saponarin) have membrane-stabilizing activity, hepatoprotective, antioxidant, and CYP2E1 inhibitory effect [41]. It is widely believed that the main component of paracetamol toxicity is CYP2E1-mediated metabolism of paracetamol to NAPQI [42]. Along with CYP2E1, other cytochrome P 450 isoforms, such as CYP1A2 and CYP3A, are also considered relevant to paracetamol toxicity [43]. Regarding cytochrome P-450-mediated metabolism of paracetamol, reducing the expression and activity of each one of the isoforms taking part in it, will lead to reduced toxicity of this compound, as we confirmed in this study. We observed that either administered alone or with paracetamol after a 7-day pretreatment period, saponarin decreased the total level of cytochrome P450, as well the activity of AH, a marker enzyme for CYP2E1 [44], and EMND, a marker enzyme for CYP3A4 [45]. According to Kumarappan et al. [41], this is probably due to direct inactivation or inhibition of enzyme expression. On the basis of our results we would speculate that acting as an inhibitor of drug metabolizing enzyme system saponarin decreased the formation of toxic metabolite of paracetamol, NAPQI, and thus decreased its toxicity.

## 6. Conclusion

From the experiments and results obtained, both *in vitro* and *in vivo,* we conclude that saponarin maintained normal biochemical parameters, mitigated the induction of oxidative stress, successfully restored liver function and architecture, and successfully alleviated hepatic damage induced by paracetamol. Possible mechanisms of saponarin protection might be reduction of free oxygen species formation by increasing the activity of antioxidant enzymes and the amount of endogenous antioxidant GSH. On the other hand, the protective effect of saponarin could be also attributed to its metabolic-mediated activities. However, further investigations should be done to estimate the appropriate dosage for this phytochemical in cases of liver damage in human. 

## Supplementary Material

HPLC chromatogram of saponarin: HPLC was performed on Shimadzu 10 Advp (Japan) chromatographic system (UV-VIS detector SPD with fixed analytical wavelengths set at 254 nm); Spherisorb C18 ODS column 5 *µ*m, 250 x 4.6 mm; mobile phases: MeOH-H2O (70:30 v/v); flow-rate: 1.5 mL/min^−1^.

## Figures and Tables

**Table 1:** Effect of saponarin (SP) and silymarin (SL) on cell viability, LDH leakage into the medium, GSH level, and MDA quantity assessed in isolated rat hepatocytes.

Group	Viability (%)	LDH (*μ*mol/min/mill cells)	GSH (nmol/mill cells)	MDA (nmol/mill cells)
Control	83 ± 3.4	0.112 ± 0.03	22 ± 1.9	0.07 ± 0.01
SP 60 *μ*g/mL	53 ± 3.8*	0.204 ± 0.05*	12 ± 0.5*	0.123 ± 0.01*
SP 6 *μ*g/mL	67 ± 3.8*	0.173 ± 0.04*	15 ± 0.5*	0.09 ± 0.002*
SP 0.06 *μ*g/mL	76 ± 1.5	0.166 ± 0.07*	20 ± 2.2	0.08 ± 0.01
SP 0.006 *μ*g/mL	77 ± 2.5	0.142 ± 0.04*	21 ± 1.1	0.07 ± 0.01
SL 50 *μ*g/mL	44 ± 1.6*	0.210 ± 0.01*	10 ± 0.5*	0.136 ± 0.003*
SL 5 *μ*g/mL	61 ± 1.1*	0.159 ± 0.03*	15 ± 1.1*	0.127 ± 0.002*
SL 0.05 *μ*g/mL	69 ± 1.5*	0.140 ± 0.06*	17 ± 1.6	0.09 ± 0.003*
SL 0.005 *μ*g/mL	79 ± 5.5	0.115 ± 0.04	18 ± 4.4	0.08 ± 0.002*

Data are expressed as mean ± SEM of four different experiments. *Significant difference from control values (Mann-Whitney *U * test, *P* < 0.05).

**Table 2:** Effect of saponarin and silymarin pretreatment on hepatic liver peroxidation and antioxidant profile in rats challenged with paracetamol.

Group	MDA^a^	GSH^a^	SOD^b^	CAT^c^	GPx^b^	GR^b^	GST^d^
Control	0.655 ± 0.05	5.52 ± 0.63	0.169 ± 0.018	121.0 ± 7.8	0.131 ± 0.016	0.222 ± 0.028	0.233 ± 0.03
PC	0.854 ± 0.04*	0.96 ± 0.05**	0.114 ± 0.012*	88.3 ± 10*	0.089 ± 0.01*	0.137 ± 0.02*	0.160 ± 0.01*
SP	0.635 ± 0.07	5.14 ± 0.31	0.158 ± 0.015	110.4 ± 16	0.125 ± 0.02	0.207 ± 0.03	0.208 ± 0.02
PC + SP	0.704 ± 0.02^+^	1.23 ± 0.15^∗+^	0.141 ± 0.02^∗+^	94.6 ± 7.8	0.108 ± 0.015	0.191 ± 0.02^+^	0.194 ± 0.02^+^
SL	0.673 ± 0.07	5.01 ± 0.64	0.162 ± 0.024	137.6 ± 13	0.144 ± 0.02	0.200 ± 0.09	0.245 ± 0.03
PC + SL	0.690 ± 0.06^+^	1.40 ± 0.12^∗+^	0.138 ± 0.02^∗+^	108.1 ± 7^+^	0.113 ± 0.01^+^	0.199 ± 0.02^+^	0.149 ± 0.03*

Treatment: paracetamol administered at a dose of 600 mg/kg i.p., alone (PC group) and two hours after the last administration of the hepatoprotective agents (SP + PC and SL + PC groups); saponarin (SP) (80 mg/kg bw/day) and silymarin (SL) (100 mg/kg bw/day were administered p.o. for 7 days. Data are expressed as mean ± SEM of six rats. *Significant difference from control values (Mann-Whitney *U * test, *P* < 0.05). ^+^Significant difference from paracetamol-treated group (Mann- Whitney *U * test, *P* < 0.05).

^a^nmol/g protein.

^b^nmol/min/mg protein.

^c^ *μ*mol H_2_O_2_/min/mg protein.

^d^nmol CDNB-GSH conjugate formed/min/mg protein.

**Table 3:** Cytochrome P450, EMND, and AH activities measured in rat liver microsomes.

Group	EMND activity nmol/mg protein	AH activity nmol/mg protein	Cytochrome P450 nmol/mg protein
Control	0.486 ± 0.032	0.075 ± 0.003	0.325 ± 0.04
Paracetamol	0.467 ± 0.02	0.078 ± 0.005	0.345 ± 0.04
Saponarin	0.371 ± 0.011*	0.047 ± 0.004*	0.244 ± 0.05*
PC + SP	0.375 ± 0.006^∗+^	0.048 ± 0.005^∗+^	0.236 ± 0.03^∗+^
Silymarin	0.340 ± 0.013*	0.051 ± 0.005*	0.255 ± 0.03*
PC + SL	0.346 ± 0.02^∗+^	0.048 ± 0.004^∗+^	0.262 ± 0.02^∗+^

Treatment: paracetamol administered at a dose of 600 mg/kg i.p alone and two hours after the last administration of the hepatoprotective agents (SP + PC and SL + PC groups); saponarin (SP) (80 mg/kg bw/day) and silymarin (SL) (100 mg/kg bw/day) were administered p.o. for 7 days. Data are expressed as mean ± SEM of six rats. *Significant difference from control values (Mann-Whitney test, *P* < 0.05). ^+^Significant difference from paracetamol-treated group (Mann-Whitney test, *P* < 0.05).
