# Supplementary material for: Hepatoprotective and Antioxidant Effects of Saponarin, Isolated from Gypsophila trichotoma Wend. on Paracetamol-Induced Liver Damage in Rats
Source: Biomed Res Int. 2013 Jun 26;2013:757126. doi: 10.1155/2013/757126 (PMC3708395; doi:10.1155/2013/757126)
Supplement: Supplementary file 1 — HPLC chromatogram of saponarin: HPLC was performed on Shimadzu 10 Advp (Japan) chromatographic system (UV-VIS detector SPD with fixed analytical wavelengths set at 254 nm); Spherisorb C18 ODS column 5 µm, 250 x 4.6 mm; mobile phases: MeOH-H2O (70:30 v/v); flow-rate: 1.5 mL/min−1. [file 757126.f1.pdf]

==== Shimadzu LCsolution Method File Infomation ====

Sample Information

Acquired by	: Admin
Sample Name	: Proba3st
Sample ID	:
Vail#	:
Injection Volume	: 20 uL
Data Filename	: Arginin 12.lcd
Method Filename	: julian.lcm
Batch Filename	:
Report Filename	: Method File Report.lcr
Date Acquired	: 18.4.2013 ז. 11:19:10
Data Processed	: 18.4.2013 ז. 11:40:26

Chromatogram

Proba3st C:\LabSolutions\Data\Project1\Arginin 12.lcd

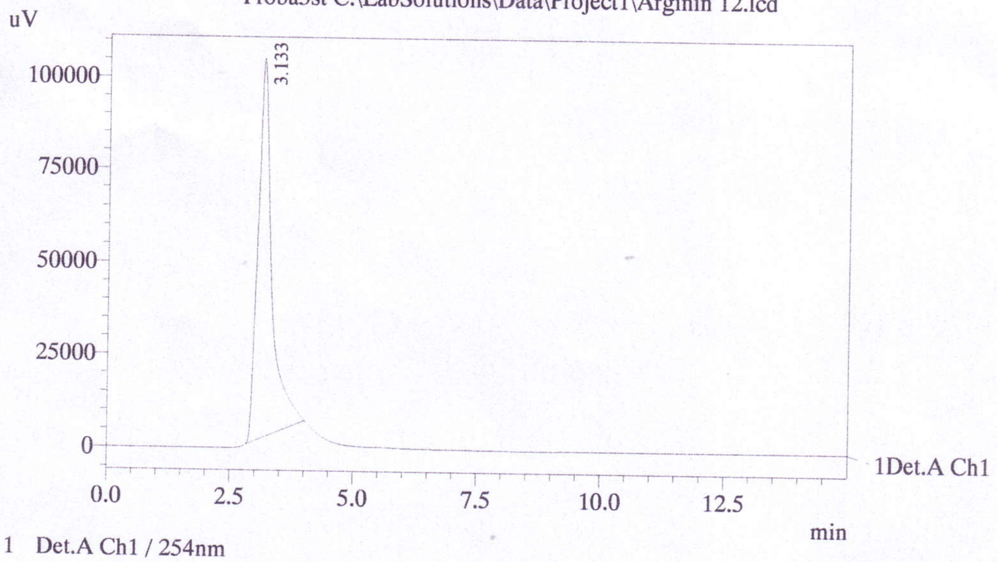

1 Det.A Ch1 / 254nm

PeakTable

Detector A Ch1 254nm

Peak#	Ret. Time	Area	Height	Area %	Height %
1	3.133	2003093	102447	100.000	100.000
Total		2003093	102447	100.000	100.000
